# Cross-cultural conceptualization of a good end of life with dementia: a qualitative study

**DOI:** 10.1186/s12904-022-00982-9

**Published:** 2022-06-08

**Authors:** Mayumi Nishimura, Karen Harrison Dening, Elizabeth L. Sampson, Edison Iglesias de Oliveira Vidal, Wilson Correia de Abreu, Sharon Kaasalainen, Yvonne Eisenmann, Laura Dempsey, Kirsten J. Moore, Nathan Davies, Sascha R. Bolt, Judith M. M. Meijers, Natashe Lemos Dekker, Mitsunori Miyashita, Miharu Nakanishi, Takeo Nakayama, Jenny T. van der Steen

**Affiliations:** 1 Department of Health Informatics, School of Public Health, Graduate School of Medicine, Kyoto University, Sakyo-ku, Yoshida Konoe-cho, Kyoto, 606-8501 Japan; 2 Dementia UK, 7th Floor, One Aldgate, London, EC3N 1RE UK; 3 Marie Curie Palliative Care Research Department, University College London, Gower Street, London, WC1E 6BT UK; 4 Botucatu Medical School, Sao Paulo State University (UNESP), Av. Prof. Mario Rubens Guimaraes Montenegro, Botucatu, SP 18618-687 Brazil; 5 Center for Health Technology and Services Research, University of Porto (ESEP/CINTESIS), R. Dr. Plácido da Costa, 4200-450 Porto, Portugal; 6 School of Nursing, McMaster University, 1280 Main Street West, Hamilton, Ontario L8S 4K1 Canada; 7 Department of Palliative Medicine, University Hospital of Cologne, Kerpener Str. 62, 50937 Cologne, Germany; 8 Department of Nursing and Healthcare, Technological University of the Shannon: Midlands Midwest, Dublin Road, Athlone, Co Westmeath N37 HD68 Ireland; 9 National Ageing Research Institute Inc., Royal Melbourne Hospital, Royal Park Campus, Gate 4, Building 8, 34-54 Poplar Rd, Parkville, VIC 3052 Australia; 10 Research Department of Primary Care and Population Health, University College London, Gower Street, London, WC1E 6BT UK; 11 Department of Health Services Research, Care and Public Health Research Institute, Living Lab in Ageing and Long-Term Care, Faculty of Health Medicine and Life Sciences, Maastricht University, P.O. Box 616, 6200 MD Maastricht, The Netherlands; 12 Zuyderland Care, Zuyderland Medical Center, Dr. H. van der Hoffplein 1, 6162 BG Sittard-Geleen, The Netherlands; 13 Institute of Cultural Anthropology and Development Sociology, Leiden University, Pieter de la Court Wassenaarseweg 52, 2333 AK Leiden, The Netherlands; 14 Department of Palliative Nursing, Health Sciences, Tohoku University Graduate School of Medicine, Sendai, Japan; 15 Department of Psychiatric Nursing, Tohoku University Graduate School of Medicine, 2-1 Seiryo-machi, Aoba-ku, Sendai, Miyagi 980-8575 Japan; 16 Department of Public Health and Primary Care, Leiden University Medical Center, Hippocratespad 21, 2333 ZD Leiden, The Netherlands; 17 Department of Primary and Community Care, Radboud University Medical Center, Nijmegen, the Netherlands

**Keywords:** Dementia, Palliative care, Culture

## Abstract

**Background:**

Research on the nature of a “good death” has mostly focused on dying with cancer and other life-limiting diseases, but less so on dementia. Conceptualizing common cross-cultural themes regarding a good end of life in dementia will enable developing international care models.

**Methods:**

We combined published qualitative studies about end of life with dementia, focus group and individual interviews with the researchers, and video-conferencing and continuous email discussions. The interviews were audio-recorded and transcribed verbatim. The data were analyzed thematically, and the researchers developed common themes referring to their original studies.

**Results:**

Fourteen qualitative researchers representing 14 cross-cultural studies covering qualitative data of 121 people with dementia and 292 family caregivers. The researchers and data were from eight countries UK, The Netherlands, Japan, Portugal, Germany, Canada, Brazil, and Ireland. Three focus groups, five individual interviews, and video-conferencing were conducted and feedback on multiple iterations was gained by 190 emails between May 2019 and April 2020 until consensus was achieved. Nine cross-culturally common themes emerged from the discussions and shared interpretation of the data of persons with dementia and family caregivers. Three represent basic needs: “Pain and Symptoms Controlled,” “Being Provided Basic Care,” and “A Place like Home.” Other themes were “Having Preferences Met,” “Receiving Respect as a Person,” “Care for Caregivers,” “Identity Being Preserved,” “Being Connected,” and “Satisfaction with Life and Spiritual Well-being.” “Care for Caregivers” showed the greatest difference in emphasis across cultures. Good relationships were essential in all themes.

**Conclusions:**

The common cross-cultural themes comprise a framework underpinned by value placed on personhood and dignity, emphasizing that interdependency through relationships is essential to promote a good end of life with dementia. These themes and valuing the importance of relationships as central to connecting the themes could support care planning and further development of a dementia palliative care model.

**Trial registration:**

The Graduate School and Faculty of Medicine Kyoto University (R1924–1).

**Supplementary Information:**

The online version contains supplementary material available at 10.1186/s12904-022-00982-9.

## Background

Dementia is increasingly recognized as a terminal illness requiring a palliative care approach especially in its advanced stages [1–4]. Understanding the perceptions of what constitutes a good end of life including a good death by people diagnosed with dementia could help improve the provision of palliative care and both quality of life and dying in this population.

Research on the nature of a “good death” has mostly focused on dying with cancer and other life-limiting diseases, but less so on dementia [5–7]. Hales et al. [5] systematically reviewed “good death” concepts in 17 studies that included the general public, patients with life-threatening diseases (cancer, heart diseases, AIDS), bereaved family, and healthcare professionals: “*physical, psychological, social, spiritual or existential experiences,” “the nature of health care”* such as teamwork, and communication about treatment, *“life closure and death preparation,”* and *“the circumstances of death”.* A recent systematic review of cross-cultural components of a good death in 29 studies of patients with cancer and some other disease such as AIDS and cardiovascular disease identified some core elements related to control, such as “*clear decision-making*” and *“preparation for death”* [6]. In countries where individualism is valued, such as USA, UK, and Canada, autonomy and independence were central to a good death [6, 7]. However, this was less so in Asian cultures [6]. Importantly, neither review included any studies about dementia. Therefore, to underpin evidence-based practice in dementia care, research is needed about what is important in achieving a good end of life in the context of loss of control as a result of dementia.

However, the course of dementia often involves specific challenges [8]. The end of life in dementia may comprise an extended period with death difficult to predict. Decision-making capability inevitably deteriorates owing to cognitive dysfunction, which negatively affects autonomy [9]. Further, people with advanced dementia often experience a variety of functional impairments and comorbidities, such as delirium, eating difficulties, and pneumonia. Overall functioning declines at the end of life, which leads to high caregiver burden [10–13]. Given these factors, a “good death” or, more extended, a “good end of life,” may be characterized by unique components on top of possible cross-cultural differences. By bringing together and synthesizing the intimate knowledge of both researchers who conducted relevant studies in different cultures and their data, we aimed to develop a shared concept of a good end of life in dementia and to identify how its components may vary across different cultures. A shared understanding of a good end of life with dementia, including Western and Eastern values, can guide policy, practice guidelines and (palliative) care models to contribute to improvement of care for persons with dementia.

## Methods

### Aim

To conceptualize common cross-cultural themes regarding a good end of life in dementia.

### Design

We conducted a study informed by a novel meta-qualitative approach, the lead researchers bringing together researchers who became our co-researchers as they had conducted original studies that had evidenced elements of a good end of life in dementia. Our novel meta-qualitative approach aimed to capture data across all the included research projects in a standardized way including an extra, layer of qualitative research with the researchers who had conducted those studies. Previous meta-qualitative approaches restricted to the analysis of transcripts or publications [14, 15]. However, combining documented findings from published studies only may not adequately capture diversity in worldviews and epistemology underlying each primary study [16–19]. In the current study, the lead researchers brought together and interviewed co-researchers from different countries about their qualitative data, which had been collected and coded as part of original studies. Involving researchers of original studies with an in-depth understanding of their data would establish rigor in the process of interpretations and synthesis of primary studies that goes beyond the evaluation of published data and allows the enrichment of epistemological views and contexts. JTS, MN, and TN with cross-culturally diverse backgrounds from Europe and Asia developed the research protocol. This study was approved by the ethics committee of the Graduate School and Faculty of Medicine Kyoto University (R1924–1).

### The study procedure

**Fig. 1:**
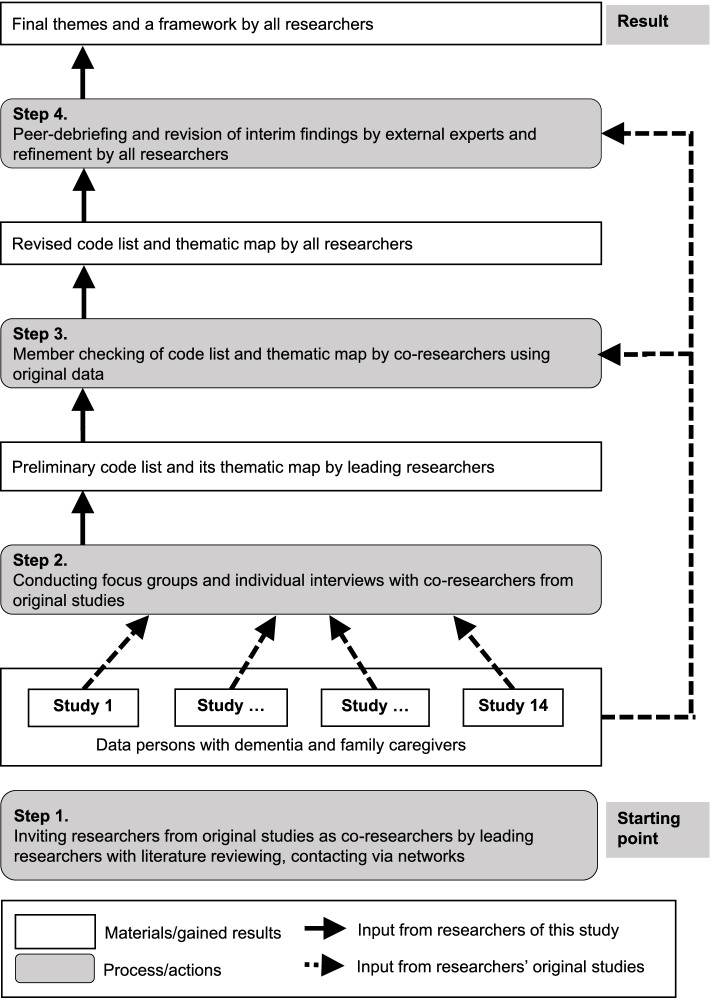
The inductive process of interpretation underpinned by communications with participating researchers and their original studies. Note: Leading researchers were the researchers who lead the study. Co-researchers were researchers who participated in this study. Researchers were the leading authors and all co-researchers involved in this study and co-authored its reporting

We identified common cross-cultural themes of a good end of life with dementia with four steps: (1) inviting researchers from original studies, (2) conducting focus groups and individual interviews with co-researchers to identify important items, (3) member checking of the code list and its thematic map by co-researchers using their original data, (4) peer-debriefing and revision of interim findings by external experts and refinement by all researchers to ensure the trustworthiness and transferability of the results (Fig. 1). Through these steps, three types of data were available for triangulation: interviews with co-researchers, their data, and peer-debriefing. JTS (Ph.D., female epidemiologist, expert in palliative care research in dementia having experience with qualitative research) and MN (a female occupational therapist in dementia care, master of public health trained qualitative researcher) were the leading researchers. We further refer to the participating researchers as co-researchers in this study.

#### Step 1. Inviting researchers from original studies as co-researchers

We employed purposive sampling, inviting researchers from diverse regions around the globe [19–21]. We intended to invite researchers who had collected, analyzed, and interpreted data on perceptions of a good end of life from the perspective of the person with dementia or family caregivers taking that perspective (therefore, not limited to professional caregiver views) or were in the process of doing so.

With hand searching for published literatures in English in recent years [19, 22, 23], researcher JTS contacted potentially eligible researchers, known through the European Association for Palliative Care and other networks, inquiring if they had qualitative data about end-of-life experiences in dementia and provided information about the project. JTS also invited 16 researchers currently undertaking studies asking to attend planned group discussions and a data synthesizing process in English.

We stopped inviting researchers after 14 agreed to participate. We considered this number would be a balance between obtaining a broad range of diverse cultural views, yet feasible to conduct focus groups [24, 25]. The findings of the 14 researchers’ studies are detailed in Table 1.

**Table 1:** Researchers’ studies

Researchers’ ID (Nation) Year of data collection	Research Aim	Design	Analysis approach	Numbers of participants (Numbers of bereaved relatives)	Main Findings
1 (Canada) 2016 (McCleary et al., 2018) [26]	Exploring family and staff experiences of end of life and end-of-life care for people with dementia	Multiple focus groups	Thematic analysis	19 family members and 77 care staff in long-term care homes (19)	Three themes emerged: “knowing the resident,” “the understanding that they are all human beings,” and “the long slow decline and death of residents with dementia.”
2 (Germany) 2013 (Schmidt et al., 2018) [27]	Identifying the needs of people with advanced dementia in their final phase of life and to explore the aspects relevant to recognize, and how to meet these needs	Multi-perspective qualitative study using grounded theory methodology conducting group discussions, individual interviews, and participant observation	Grounded Theory	42 health professionals, 14 relatives, and 30 residents observed at nursing homes (0)	Physical needs: “food intake,” “physical well-being,” and “physical activity and recovery.” Psychosocial needs: “adaptation of stimuli,” “communication,” “personal attention,” “participation,” “familiarity and safety,” and “self-determination.” Spiritual needs: “religion.” Results revealed a multitude of key aspects and stressing the importance of personhood.
3,4 (UK) 2012–2014 (Moore et al., 2017) [28]	Understanding the experiences of caregivers during advanced dementia, exploring the links between mental health and experiences of end-of-life care	Mixed methods of a longitudinal cohort study and individual interviews	Thematic analysis	6 family caregivers at home and 29 at care homes (12)	For family caregivers three main themes emerged. “Importance of relationship with care services,” “understanding of the progression of dementia,” and “emotional responses to advanced dementia.” Family caregivers’ ability to control and influence end-of-life care was overarching.
4,5 (UK) 2012–2013 (Davies et al., 2017) [29]	Exploring the views of family caregivers about quality end-of-life care for people with dementia	Using in-depth interviews analysed using thematic analysis Purposive sampling from a third sector organisation’s caregiver network was used to recruit	Thematic analysis	47 family caregivers including one recently diagnosed, 14 currently caring, and 32 bereaved family caregivers (32)	Quality end-of-life care for people with dementia is perceived as “fostering respect and dignity,” and “showing compassion and kindness.”
4,6 (UK) 2009–2010 (Harrison Dening et al., 2012)[30]	Exploring whether people with dementia and their caregivers were able to generate and prioritize preferences for end-of-life care	Nominal group technique	Thematic content analysis	17 interviewees; 6 people with dementia,5 caregivers, 6 dyads of people with dementia and family caregivers (0)	“Quality of care,” “family contact,” “dignity and respect” were ranked as significant themes by all groups. Analysis of transcripts revealed three main themes: “Quality of care,” “independence/control,” and “care burden.”
6 (UK) 2013–2016 (Bamford et al., 2018) [31]	Identifying key components of good end-of-life care for people with dementia and to inform a new intervention	Semi-structured interviews, focus group interviews, discussions, and observations of routine care	Thematic analysis prior to integrative analysis which resulted in key themes across stakeholder groups	259 interviewees; national experts, service managers, care staff, people with dementia, family caregivers, health care professionals (12)	Seven key factors were required for the delivery of good end-of-life care: “timely planning and discussions,” “recognition of end of life and provision of supportive care,” “coordination of care,” “effective working relationships with primary care,” “managing hospitalisation,” “continuing care after death,” and “valuing staff and ongoing learning.”
7 (Ireland) 2014–2015 (Dempsey et al., 2018) [32]	Exploring the experiences of caregivers who provide end-of-life care for a person with late-stage dementia at home	Semi-structured interviews were conducted with current and past family caregivers	Data was analysed using interpretative phenomenological approach	17 current family caregivers and 6 past family caregivers of persons with dementia living at home (6)	Four super-ordinate themes were identified which described the challenges faced by caregivers at different stages of their caregiving journey. “The experience of dementia grief,” “parenting the parent,” “seeking support,” “death, dying and life after death.”
8 (Portugal) 2015 (Lillo-Crespo et al., 2018) [33]	Identifying the strengths and weaknesses in daily life perceived by people with dementia and family caregivers in seven European countries	22 in-depth qualitative case studies were completed in seven European countries across a range of care settings considered typical within that country	Case study method, a constant comparative method with thematic synthesis	56 interviewees; 22 relatives,13 health care professionals, and 21 persons with dementia in 8 Scottish, 9 Spanish, 6 Swedish, 6 Finnish, 7 Slovenian, 7 Czech Republic, and 13 Portuguese (0)	Identified themes were “Early diagnosis,” “good coordination between service providers,” “future planning,” “support and education for family caregivers,” “enabling the person with dementia to live the best possible life,” and “education on advanced dementia for professional and family caregivers.”
9,10 (Japan) 2017 (Watarai et al., 2019) [34]	Identifying what the components of the good death with dementia are and what the common components or individual components for people with dementia, family caregivers, and medical professionals are. To explore different views between the three groups.	Semi-structured interviews were conducted with current and past family caregivers	Thematic content analysis	10 people with mild cognitive impairment, 10 family caregivers, 3 physicians, 4 nurses, 6 care workers (0)	“Maintaining dignity,” “Natural care,” “Family relationships” were common categories among three groups and found over 70% of frequency in each group while “Proper medical care,” “Familiar environment,” “Economic power” were different priorities among these groups.
11 (Japan) 2016 (Nishimura et al., 2020) [35]	Conceptualising a good end of life for people with dementia from the perspectives of bereaved family caregivers in Japan	A qualitative study using in-depth, semi-structured interviews focused on the family caregivers’ perceptions of their loved one’s experiences	Thematic analysis	30 bereaved family caregivers (30)	A good end of life for people with dementia means experiencing death as “Peaceful” while “Personhood” is being maintained at a “Comfortable Place” allowing for feelings of “Satisfaction with life.”
12,13 (Netherlands) 2018 (Bolt et al., 2019) [36]	Investigating loved ones’ experiences with end-of-life care for people with dementia, and compare the nursing home and home setting	Individual, in-depth, semi-structured interviews with loved ones	Thematic analysis, inductive and deductive coding; critical realist approach	32 bereaved family caregivers of people with dementia; 8 from homes, 24 from nursing homes (32)	The person behind the disease being acknowledged by nursing home staff. The end-of-life experience of the person and care role of the family is different between nursing home and home care. (Surrogate) end-of-life decision making raised similar challenges in the nursing home and home setting. Nursing home and home care professionals should properly inform loved ones of people with dementia about the disease and end-of-life trajectory as this may encourage confidence in decision making even in the case of unknown patient wishes.
12,13 (Netherlands) 2019–2020	Investigating the thoughts of people with dementia about care now and in the future and discussing wishes for the end of life	Individual interviews with people with dementia	Inductive content analysis	17 people with dementia (0)	For the persons with dementia, it was important to live a meaningful life until the end and to be acknowledged as a unique individual. They placed a lot of trust in others to take care of them or to make decisions for them. Although thinking about their future or the end of life was unsettling or frightening for some, most also showed acceptance and contentment with life.
14,15 (Netherlands) 2014–2015 (Lemos Dekker, 2018)[37]	Exploring how people with dementia, their family members, and professional care workers manage the end of life with dementia	Ethnographic study, 18 months of fieldwork, in-depth interviews and focus groups	Thematic analysis	40 interviews with family members, observation people with dementia, and focus groups with professional caregivers	Death was often welcomed by family members as they experienced it as a form of relief, and thus it can be considered as a form of care.
6, 16 (Brazil & UK) Ongoing at 2020	Understanding what people with dementia consider a good death in light of their dementia diagnosis?	Semi-structured interviews	Thematic content analysis	People who have a diagnosis of dementia (of any type), being aware of their diagnosis, and have the capacity to participate in a semi-structured interview (0)	Data collection is ongoing.

#### Step 2. Conducting focus groups and individual interviews with co-researchers from original studies

The leading researchers JTS and MN convened three focus group discussions between 10th and 23rd May 2019 with the co-researchers who prepared for the discussions. Two were held face-to-face during international conferences, and one was conducted through video conferencing. Discussions were moderated by JTS. Notes were made during and after the interviews by JTS and MN. All interviews were closed meetings, audio-recorded, with all co-researchers giving written informed consent.

The topic guide was developed from co-researchers’ studies and a pilot discussion [24]. The main questions were (1) “What are good end-of-life experiences based on your data?” and (2) “What are the similarities and differences in comparison with other data (studies)?” (See Additional file 1 published as supplementary material). All co-researchers attended one of two available focus groups addressing the same topics, and all but one participated in the third, follow-up focus group. Subsequently, MN conducted individual interviews (*n* = 4) for more in-depth exploration of themes as needed, such as the role of religion in selected studies.

#### Step 3. Member checking of the code list and its thematic map by co-researchers using original data

Member checking is also called respondent validation or participant validation and it is a process of active involvement of research participants in checking and confirming preliminary results of the research. To reduce potential researcher bias, interviews or analyzed data are returned to a study participant [38]. In our study, we used member checks of focus groups and member checks of synthesized analyzed data. The two leading researchers circulated preliminary analyses shortly after focus groups and repeatedly afterward showing the code list and a thematic map to co-researchers for member checking. The co-researchers checked the code list for consistency using their original data codes. To identify common and different items across the studies, the co-researchers verified each item to determine its fit to their data and cultural context by choosing from the following criteria;

- Yes, I found it (in my data), and it fully fits the data (study population)
- Yes, I found it, but it fits the data (study population) only in part
- No, I did not find it, but it may fit my data (study population)
- No, I did not find it, and it does not fit the data (study population)

The co-researchers left comments on the list why it would or would not fit their data or population. Based on the feedback, the leading researchers reworded, replaced, or merged the codes, and revised the initial thematic map.

#### Step 4. Peer-debriefing and revision of interim findings by external experts and refinement by all researchers

We additionally asked external experts to review the code list and the thematic map resulting from Step 3. Based on their feedback, the revised thematic map was returned to co-researchers. All members commented on this via email and the leading researchers made further amendments. All co-researchers were involved throughout the process and ultimately agreed with the final version of concepts of a good end of life with dementia and the thematic map.

### Data analysis

We analyzed all interview transcripts, notes, feedback, and comments through email exchange using of thematic analysis by Braun and Clark [39, 40]. MN transcribed individual interviews and used a professional transcribing service for the international meetings. MN read the transcripts repeatedly and coded data inductively focusing on the important aspects of a good end of life with dementia. Categories were constructed by all available codes, capturing meta meaning of codes. The code list was revised with the constant comparative method. JTS reviewed and revised, discussing with MN about the open coding and categorizing process. MN referred to co-researchers’ published papers and added possibly important additional concepts to a preliminary code list. Subsequently, all researchers discussed the labels of the code list and the thematic map throughout member checking and peer debriefing until consensus was reached [38, 41].

In the analyses, we avoided broad concepts such as spirituality and dignity. Instead, we tried to incorporate all important concepts-framed positively-using the more concrete descriptions in lower-level categories. We managed the transcriptions using Microsoft Excel® 2016 to scrutinize inductive coding by all researchers.

## Results

Of 16 researchers contacted, 14 (88%) agreed to participate. Two declined because they could not attend the scheduled focus groups owing to schedule clashes. Table 2 shows the co-researchers’ characteristics. We used data based on 14 projects, which included interview and observation data from a total of 121 people with dementia and 292 family caregivers. Thirteen projects finished data collection. Of those, 12 were published [26–37]. One study was in the process of interviewing when we conducted the study.

**Table 2:** Characteristics of co-researchers (*n* = 14^a^)

Age category	
20–35 years old	3
36–50 years old	8
51 and up	3
Gender	
Men	4
Women	10
Background	
Clinical	
Nurse	6
Physician	3
Psychologist	2
Not clinical	
Sociologist	1
(Researcher only)	
Anthropologist	1
Epidemiologist	1
Academic qualification	
PhD	12
Master	2
Nation	
United Kingdom (UK)	4
The Netherlands (NL)	3
Japan (JP)	2
Portugal (PT)	1
Germany (GM)	1
Canada (CA)	1
Brazil (BZ)	1
Ireland (IL)	1
Being a bereaved relative of someone with dementia (personally)	
yes	9
no	5

^a^Not including the 2 leading researchers

Three focus groups and five individual interviews were held with a median duration of 75 minutes (range: 70–89 minutes) with no further topics raised at the closing of every interview. In step 3, all co-researchers received 87 codes referring to their data and provided feedback as to whether categories and themes were or were not true to their data.

In step four, peer-debriefing, the initial thematic map was a pyramid shape, indicating “Pain and Symptom Control” as a prerequisite for all other “good death” themes (Additional file 2). The thematic map was pointed as skewed towards a Western view by an external expert (a Japanese palliative care expert). The majority of co-researchers in our research team were from Europe; hence, we asked Asian external practitioners (a Japanese dementia care nurse and a Buddhist monk with international hospice experience) to review and discuss Asian views to obtain an additional independent perspective. The use of opioids in the terminal phase of people with dementia is not usual in Japan, so the Japanese practitioners suggested a circular arrangement that did not premise “Pain and Symptom Control.” The circle also allowed for visualizing a central position for relationships. After we received their suggestions, the leading researchers and four co-researchers from the Netherlands and Japan discussed differences of cultural views. Next, the revised thematic map was returned to all co-researchers asking it fitted with each study’s culture (detail of peer-debriefing group discussion is provided in Additional file 2 published as supplementary material online).

**Fig. 2:**
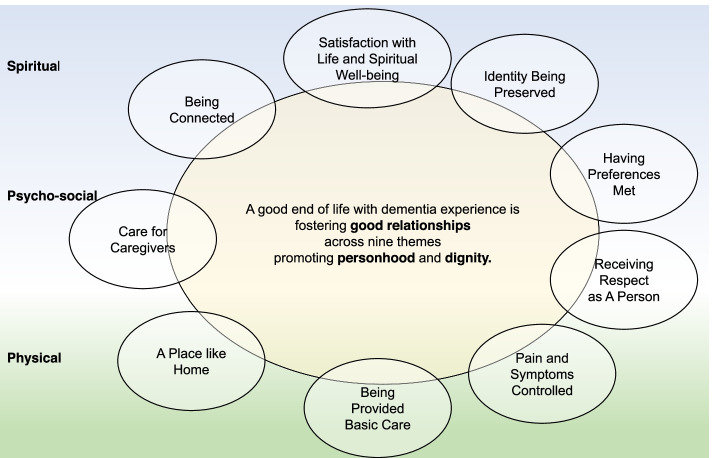
Thematic map of a good end of life with dementia

Finally, we generated a thematic map covering nine themes comprising 36 categories from 45 codes. Email discussions (in 190 emails) continued until April 2020 after which all agreed on the results. Table 3 shows the nine themes that emerged as common important aspects of a good end of life with dementia: Pain and Symptoms Controlled; Being Provided Basic Care; A Place like Home; Care for Caregivers (which means not being a burden to others); Identity Being Preserved; Having Preferences Met; Being Connected; Receiving Respect as A Person; and Satisfaction with Life and Spiritual Well-being. Figure 2 shows how these themes interrelated. All themes included categories about relationships as key to promoting personhood and dignity at the end of life. Below we discuss the themes and any cross-cultural differences.

**Table 3:** Important aspects of a good end of life with dementia.

**Themes and Categories**	**Codes (examples of important items)**
**Pain and Symptoms Controlled**	
Comfort care provided	Care aimed at maximising feelings of comfort
Physical symptoms controlled	Controlled pain and burdensome symptoms such as difficulty breathing, confusion, bedsores, contractures etcetera that cause discomfort
Function preserved	Maintenance of function
Special needs addressed	Identifying discomfort
	Balanced treatment, avoiding overtreatment or undertreatment
**Being Provided with Basic Care**	
Maintenance of hygiene	Physical hygiene, clean clothes, clean environment
Timely support	Being helped at the right time, when the person has needs, for example, to be supported when wanting to go to the toilet
Continuity of care	Remaining in the same place of care, avoiding changing care provider, information about the person is shared to make sure care processes continue smoothly
**A Place like Home**	
Familiarity with environment, people and care	Sense of familiarity in the place, familiar atmosphere with friendly people, alignment with what person is used to, such as similarity of environment, system, routines and devices
	The person accepts, adapts to the last place
	Avoiding transfer to elsewhere such as hospital or emergency room
Comfortable environment	Individual room/personal space, quiet room, relaxed/peaceful environment, enough space, free to go out for fresh air
Preferred place of care	Having opportunities to choose the last place
	Staying at home
Availability/access	Availability of nursing home if needed
**Having Preferences Met**	
Personal preferences being considered and addressed	The person's spiritual and religious preferences are respected and met
	Not being forced to do something unpleasant
Receiving support in discussions about decisions	Opportunity and support for discussions to make decisions
Having an attorney	Having a proactive, trustworthy, well-known attorney
Care planning consistent with wishes	The care is being provided based on the person's wishes
Preference being prioritized	The preference of the person* is being prioritized over family preferences*This includes a preference to protect family and have them decide
**Receiving Respect as a Person **	
Being paid attention to	Being paid attention to
Being treated attentively	Being treated carefully, the person feels no fear, for example, through gentle care such as the person being spoken to with respect, the caregivers explaining to the person what they are doing while providing care, protecting privacy
Being treated with equity	Being treated in an equal way as a person without dementia (equity)
Being treated by trustworthy caregivers	Being cared for by trustworthy caregivers
Allowed freedom	Free from physical restraints
**Identity Being Preserved**	
What the person looks like	The person looks similar as before through wearing clothes that fit the style the person wore in the past
	The person's hair is groomed in a way that fits the person’s style
How the person is treated as an individual	The person gets daily support to keep her/his image
	The person is treated in a way that fits with his/her personality (personalised care)
How the person spends time	The person keeps his/her personal daily routine
How the person reacts	The person behaves as she/he was
	The person is able to recognize the faces and names of his/her family
**Being Connected**	
Keep preferred stimulation	The person receives gentle touch according to their needs
	The person feels joy to taste, communicate; enjoys music, peaceful sounds, voice of family, the beauty of nature
Sense of connection with others	The person keeps relationships with familiar people
Family/familiar people present at the time of dying	The person is surrounded by family/familiar people (may include priests) when she/he dies
**Care for Caregivers (Not being a burden to others)**	
Caring for caregiver’s emotional and psychological well-being	People with dementia wanting to avoid emotional or psychological distress in caregivers
Caring for the caregivers’ finances	Not being a burden financially
Caring for caregivers’ health	Independent self-care as long as possible to minimise care burden or stay at home
**Satisfaction with Life and Spiritual Well-being**	
Feeling peaceful	The person feels a sense of comfort, peace, safety, and sometimes pleasure (spiritual/psychosocial comfort)
Sense of being protected	The person feels close to a higher presence (i.e., God, Allah, Angels, Saints, Hotoke)
Acceptance of life closure	The person accepts the time of his/her death
Valued as a person	The person is loved
Living with hope	The person has hope for the people around, narratives of life retained as a legacy to others, give something to others, to contribute to others

## Findings

### Themes with codes common to all data

#### Pain and symptoms controlled

Having “Pain and Symptoms Controlled” is essential for physical functioning and supports all other themes. Nevertheless, it is insufficient in and of itself because if achieved while psychosocial and spiritual needs are not met, people with dementia may not experience a good end of life.

Listening to residents, they definitely didn’t want some symptoms pain, dyspnea, and those kinds of things, quite concerned about that, particularly pain. But then you talked further this notion was kind of psychosocial comfort as well. (CA)

Maintaining function is deemed to be relevant until the end of life as much as possible from the perspective of family caregivers because being able to eat, being able to smile, being able to talk is valued at the end of life to meet the persons’ psychosocial needs. (JP)

“Pain and Symptom Controlled” affected dignity if it was ignored due to a lack of compassionately or empathically caring about the other and good relationships. People with dementia often experience difficulty communicating, so caregivers’ attention to persons’ health status affects dignity.

I often saw the pain was also connected to dignity. So, if the person was suffering or in pain, family members would often say this is not a dignified end of life, yes. (NL)

Cross-cultural difference: The category “function preserved” in this theme was not emphasized in a study from the UK. Further, perceptions on the use of opioids at the end of life differ between the cultures. It is perceived as usual in the Netherlands but exceptional in Portugal, Brazil, and Japan. In Ireland, Canada, and Germany, people receive opioids occasionally.

#### Being provided basic care

Providing basic care is regarded as default; however, it is not always provided to people with dementia. Seamless care is often an issue with changes of care providers or place of care. Providing basic care to highly dependent people for a long end-of-life period is difficult for those involved. People with dementia sometimes experience unclean environments and unhygienic conditions.

It usually was about compassionate care, being clean, loved ones around them, all of those sorts of things. (UK)

Cross-cultural difference: Basic care is different in each country. For example, soaking in a bath is considered standard care in Japan, even at the end of life while it is not generally considered basic care in European countries.

#### A place like home

People with dementia are usually sensitive to environmental changes. Being in a familiar, preferred place and comfortable environment with familiar people was commonly regarded as important at the end of life across countries.

Cross-cultural difference: Social acceptance of nursing home residence differed across cultures. In the Netherlands, the UK, Canada, and Germany, society considers spending the end of life in a nursing home as the best choice if home is not possible, or at least, as normal practice. However, in Ireland, Portugal, and Brazil, family care is emphasized, and deaths in institutions are negatively perceived. In Japan, some families prefer hospitals to provide “all possible treatments.”

#### Having preferences met

This theme was particularly important for a good end of life because people with dementia often have difficulties in retaining autonomy due to cognitive decline. Personal preferences should be considered across all themes.

They wanted to have their wishes regarding end of life adhered to. We don’t have advance care directives and dignity law yet. And they haven’t had the family carers have the discussion with the person with dementia regarding their end-of-life preferences. And it was one of the things that came up with the regret of not having had that conversation at the time. (IL)

This was complicated by uncertainty about the person’s preferences and a possible trade-off between personal preferences and “Care for Caregivers.” Retaining good relationships may promote “Having Preferences Met” because this facilitates awareness of the person’s preferences and wishes.

Cross-cultural difference: Across the countries, there is a shared ideal to prioritize the person’s preference, however, different meanings of person’s preferences are discussed. In Japan, spontaneous expressions of end-of-life wishes are appreciated, but making people with dementia decide is considered as putting pressure on them and causing anxiety. When the capacity to express preferences is limited, family is considered best positioned to understand the person’s preferences, and family preferences are often prioritized. In Brazil, people with dementia frequently value family caregivers’ wishes as their own wishes, out of concern to express wishes while depending on others is not compatible with their sense of dignity. Also, persons with dementia may perceive going with family’s decisions is perceived as a positive means to protect their families from caregiver burden. People in the Netherlands also value important relationships over full achievement of their own wishes.

#### Receiving respect as a person

Receiving respect as a person means being paid attention, being treated attentively, being treated the same as a person without dementia, being free from physical restraints, and being treated by trustworthy caregivers (Table 3). Persons with dementia require assistance with most activities of daily living, including communication, which may threaten dignity. People with dementia in care facilities in any country may feel like they are treated as objects. Compassionate, person-centered, and dignified care all promote quality at the end of life and are associated with relationships.

I think that comes out with a sort of dignity and respect thing that they [person with dementia] wanted to be treated in a certain way and not treated [as] things or treated an object. (UK)

Cross-cultural difference: In Canada, this theme is important in the context of divergent populations such as LGBT. In Japan, receiving “all possible treatment” is perceived as respectful for some people; and doing “nothing” for a dying person as a lack of respect.

#### Identity being preserved

Loss of identity, for example, through changing personality, loss of abilities, roles, or companions, is a specific theme in dementia progression and involves spiritual and existential issues. Even if an individual’s sense of identity is threatened, maintaining the daily appearance and personal routines, understanding the person’s past, and being and appreciating the person they have been, can improve their end-of-life experience.

From a clinical perspective, we have lots of complaints around end-of-life care from families. And they don’t say “Oh, my relative’s spiritual beliefs were not taken into account and they died.” You don’t even use that terminology. But what you hear, they say is… things like “[He] didn’t even look like my dad anymore” you know. That was important [and] wasn’t taken into account for him. (UK)

Cross-cultural difference: In countries where home care is dominant, preserving identity does not take as much effort and is taken for granted because care is provided by families who know the person’s history.

#### Being connected

People with dementia tend to lose social relationships and social interactions, which leads to fear and isolation including at the end of life when people are not mobile. Retaining a sense of connectedness reduces fear and isolation. Sensory stimulation such as hearing, being touched, being in social contexts, experiencing nature and a sense of transcendence can help people with dementia feel connected with the environment and their inner self.

Because they [persons with dementia] can feel compassion and they want the connectedness with others to make contact. (NL)

Cross-cultural difference: In Japan, being connected means mainly family bonds and presence, while the presence of priests is rare. In the Netherlands, Ireland, Portugal, and Brazil, this was also perceived as a religious experience in people with religious beliefs.

### Themes with initially divergent codes

#### Care for Caregivers (not being a burden to others)

People with dementia generally did not intend to be a burden for caregivers in a physical, psychological, economical, or other way. Not wanting to negatively impact on others can be a social but even so a spiritual or existential issue, which may prompt people with dementia to long for death; however, social, and other support systems can reduce the negative impact on caregivers.

Cross-cultural difference: The theme name was discussed and revised multiple times. This theme generated different solutions cross-culturally. In Japan, entering a nursing home was seen as a solution to relieve caregiver burden. This theme could be a reason to request euthanasia in the Netherlands. In Brazil, people tended to offer considerable leeway in their wishes by prioritizing family life.

They [people with dementia] would like the preferences of care at the end-of-life to be acknowledged, they would give their family members total freedom to change whatever decisions they have made if the new decisions would decrease the family's suffering. (BZ)

#### Satisfaction with life and spiritual well-being

A person’s sense of self, acceptance, pleasure, and feelings of peace were deemed the components of ‘Satisfaction with Life and Spiritual Well-being.” The theme’s codes, such as “Acceptance of life closure” relate to “Satisfaction with life” and is usually part of spiritual themes. This theme was enhanced by relationships, in particular, a person’s “Sense of being protected” by God or others whom they relate to.

Even when they are in very severe advanced dementia, sometimes, when they are connected with people who support their spirituality, sometimes, they can remember things. Sometimes, they can remember names. (PT)

Cross-cultural difference: “Being Connected” with God contributes to “Satisfaction with Life and Spiritual Well-being” for religious people, while for non-religious people, “Being Connected” with family or “Identity Being Preserved” contributed to this theme.

## Discussion

### Main findings of the study

We presented nine cross-culturally shared themes to help achieve a good end of life with dementia, integrating findings from qualitative studies in eight countries by focus group and individual interviews and de-briefing with researchers about their original data. The mapping of themes also delineated themes and issues that are relevant in some cultures, and showing the breadth of what can be important in a good end of life with dementia can help identifying what is important for individuals.

A key finding is the benefit from and value of relationships over full autonomy, even in Western countries. Relationships were discussed as a reason or promoting factors in all nine themes for people with dementia at the end of life who partially or fully depend on others to address physical, psychosocial, and spiritual aspects. The thematic map indicates that maintaining a good relationship may promote other themes in a good end of life, acknowledging there are trade-off situations when a good end of life cannot be achieved in full, or for both the person with dementia and their caregivers. Previous conceptualizations of a good death have identified autonomy or independence as central; the ability to choose freely, independently of others’ control [42–44]. The autonomy-centered model reflects the values of people in Western countries [45, 46]; however, choices and wishes are usually affected by relationships [44, 46, 47]. In our discussions and data, autonomy was rarely a central theme, neither with respect to wishes to hasten death, nor in the more extended end-of-life phase. Instead, it was about value of relationships with caregivers, family, and other familiar people. Relationships were a key part of all nine themes as people with dementia at the end of life partially or fully depend on others to address physical, psychosocial, and spiritual aspects.

Another finding was a prominent theme in dementia as “Identity Being Preserved” cross-culturally, while most conceptualizations of a good death in cancer and other chronic illnesses emphasizes “Autonomy” or “Being in Control” [26, 27, 29, 48–52]. The experience or fear of losing one’s identity may be enhanced with conditions involving declining cognition. Reviews of needs in advanced dementia, not about end of life specifically, have also indicated the importance of identity and personhood [27, 29, 53, 54]. Identity was more of an issue in residential settings than home-care settings, where identity was maintained at the end of life as a matter of course. Even if an individual’s sense of identity is threatened, our code trees suggested that the environment and good relationships, and caregivers’ attention to personal histories, maintaining appearance and routines, may support their identity.

The themes with initially most divergent codes were “Care for Caregivers” and “Satisfaction with Life and Spiritual Well-being.” “Care for Caregivers” was often discussed as avoiding caregiver burden. People with dementia may leave decision making to their family members to avoid burdening others. Since sometimes there is a trade-off between care for the caregiver and the person’s wishes [3], if the person does not advocate for their own wishes, we need to consider whether prioritizing caregiver interests is really in line with their voluntary choice. “Satisfaction with Life and Spiritual Well-being” related to spirituality more broadly than just religion, as spiritual pain is also experienced by people without specific religious beliefs. Puchalski et al. [55], in a consensus study, defined spirituality in terms of “seeking and expressing meaning and purpose” and “experiencing connectedness to the moment, self, others, nature, and the significant or sacred”. Our discussion was consistent with this definition, and the theme “Being Connected” can result in “Satisfaction with Life and Spiritual Well-being” in religious people. For people without religious beliefs, “Identity Being Preserved” was emphasized, which reflects “connected to self and others” in Puchalski’s definition. With dementia specifically, people become dependent and relationships with family and familiar surroundings are important; therefore, there may be less difference between Western and other countries in the case of dementia compared with other diseases.

### Strengths and weaknesses of the study

Participants were from diverse cultural and (non)religious backgrounds and countries. We used data from those living in institutional settings and those living at home. Diverse data enabled us to discover major commonalities and smaller differences in relevant themes of good end of life.

While traditional designs of integrating qualitative studies use published papers only, the focus group discussions were useful to identify unwritten aspects about co-researchers’ studies, increase understanding of the relationships among themes and jointly interpret commonalities and differences. Continued email discussions with the co-researchers of original studies enabled repeated refinement of themes to ensure trustworthy labelling reflecting nuance in raw data, including through recalling observations in ethnographic fieldwork, from intimate knowledge of the co-researchers.

We have drawn on the data of studies conducted before the COVID-19 pandemic. During the COVID-19 pandemic, public perceptions about a nursing home as a “Place like Home” may have changed to less favourable perceptions through social distancing and infection control measures. In the UK, where nursing homes are a common place of death for a person with dementia, more people with dementia died in private homes than before the pandemic [56]. Through the pandemic, nursing homes might be viewed as a place that limits relationships in the end-of-life period.

This study has some limitations. Although we tried to use purposive sampling, most invited co-researchers were from European regions as the research field is not fully explored in diverse regions. We, therefore, invited Asian external experts in the peer-debriefing. For the same reason, our findings about relationships might not generalize to the USA, where control and autonomy are strongly valued. Also, much of the data was obtained from family caregivers, therefore, family perspectives even though from the viewpoint of a good end of life for their relative, may influence the results.

For clinical practice, the thematic map suggests a framework of important elements of a good end of life for people with dementia. The framework acknowledges the importance of relationships as central to connecting the themes to support advance care planning (proposing a “relationship-valued” perspective in contrast to, or to complement an “autonomy-valued” perspective). Advance care planning is recommended early for people with dementia because of the declining ability to express their preferences and wishes as the disease progresses [57]. Although not all categories obtained from the qualitative data will be important for all individuals, the themes and categories may help a person, care staff, and families through suggesting what may be most important for an individual. Simultaneously, the framework emphasizes the importance of good relationships which may support a relationships-valued view in conversations about current or planned care.

Further research should examine how to use the framework in case of conflicting preferences regarding themes. It may not be feasible to optimize all themes to achieve a good end of life. For example, there may be a trade-off between “Care for Caregivers” and “Having Preferences Met.” It is important not to increase feelings of guilt by increasing pressure on caregivers to achieve difficult goals, particularly about maintaining care at home [58]. Also, we consider that some of these themes should add to palliative care model for people with dementia. The European Association for Palliative Care sought to define optimal palliative care in dementia based on a Delphi study involving experts from 23 countries [3]. According to the care goals model in the white paper, maximizing comfort is the highest end-of-life priority. In a future palliative care model, our findings suggest refining psychosocial and spiritual goals at the end-of-life phase.

## Conclusions

This study identified nine common themes of a good end of life for persons with dementia in diverse countries. Having good relationships appeared a key aspect of a good end of life. Our suggested relationships-valued framework may help to personalize care plans in end-of-life conversations.

## Supplementary Information

**Additional file 1.** Topic guide of focus groups

**Additional file 2.** Peer-debriefing discussion about initial thematic map

## Data Availability

The raw data collected during the current study are not publicly available because the dataset cannot be anonymized in such way that the participants are unrecognizable. Any requests for sharing codes and analyses should be directed to JTS and MN within 10 years from 2020.
