# Supplementary material for: Cross-cultural conceptualization of a good end of life with dementia: a qualitative study
Source: BMC Palliat Care. 2022 Jun 8;21:106. doi: 10.1186/s12904-022-00982-9 (PMC9175529; doi:10.1186/s12904-022-00982-9)
Supplement: Supplementary file 1 — Additional file 1. Topic guide of focus groups [file 12904_2022_982_MOESM1_ESM.docx]

| **Additional file 1.** Topic guide of focus groups | |
| --- | --- |
| Focus group 1 and 2 |  |
| *Before the meeting* | The leading researchers asked the co-researchers to review their codes and findings about a good end of life for dementia. In case the data comprised professional caregivers’ perspectives too, the leading researchers asked the co-researchers to single out perspectives from the person with dementia or the family caregiver reflecting on the perspective of the person^a^. |
| *Topic 1.* | Could you write down what good end-of-life experiences for people with dementia are, based on your data? Please prioritize the three most important items. |
|  | What is a bad end-of-life experience for people with dementia? Are there any other important issues? |
| *Topic 2.* | What are the similarities and differences of findings in comparison with other data (studies)? |
|  | Do you feel these are country-specific? |
| Focus group 3 |  |
| *Before the meeting* | The leading researchers circulated a preliminary analysis from the previous two focus group sessions among the co-researchers |
| *Topic 1.* | How do you feel about the preliminary analysis from the previous two focus group sessions? |
| *Topic 2.* | Are there any other important themes? |
|  | Do you feel there are themes that are culturally specific?^a^ |
| **^a^** We also intended to address care goals and possible adaptations to the EAPC dementia white paper figure (van der Steen et al., 2014) in the third focus group session, but the discussions did not suffice due to time limitations in the face-to-face group interview. | |
