# Supplementary material for: Cross-cultural conceptualization of a good end of life with dementia: a qualitative study
Source: BMC Palliat Care. 2022 Jun 8;21:106. doi: 10.1186/s12904-022-00982-9 (PMC9175529; doi:10.1186/s12904-022-00982-9)
Supplement: Supplementary file 2 — Additional file 2. Peer-debriefing discussion about initial thematic map [file 12904_2022_982_MOESM2_ESM.docx]

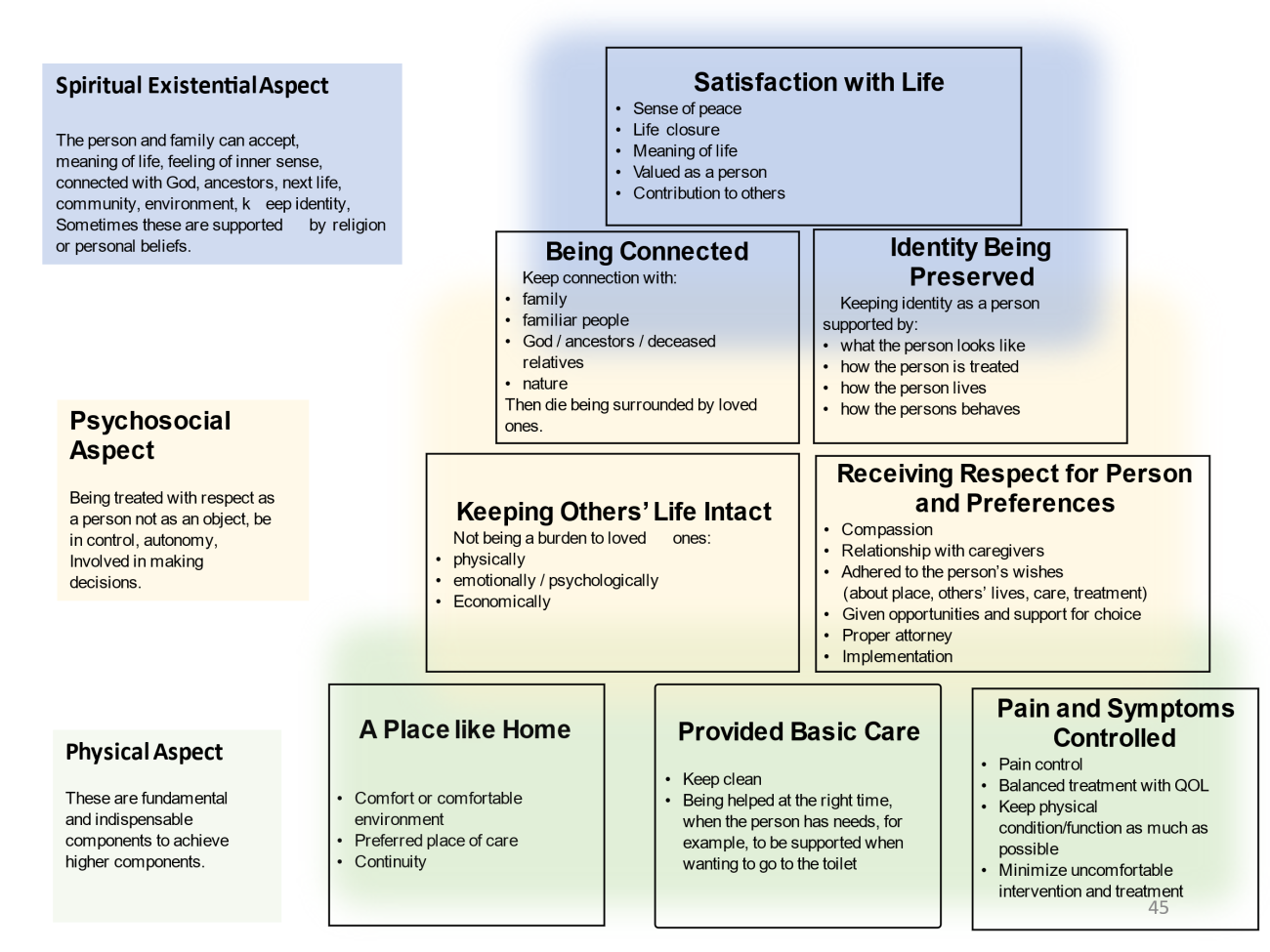
Additional file 2. Peer-debriefing discussion about initial thematic map

Our initial category map had each theme arranged in a pyramid shape, with “Pain and Symptoms Controlled” at the bottom. The shape explained that “Pain and Symptom Controlled” is the premise for all the other good death themes. The first external expert pointed that this was due to European values, where there is less hesitance to use opioids in clinical practice.

In Asia, including Japan, there is a certain degree of hesitation to use opioids in dementia deaths, except for cancer deaths. From a clinical experience, a person with dementia dies in a sleepy state with minimal respiratory distress with a gradually diminishing intravenous drip for hydration, without the use of opioids. Also, if the pain was at an acceptable level, and if some other important themes are well achieved, people may consider the experience over all good end of life. Thus, the arrangement of the themes was changed from a pyramid to a circle and returned to our co-researchers.

The circle shape also provided insights into “relationships” as a central role and meaning with all themes. The co-researchers discussed that each good EoL theme had categories relating to “relationships.” Furthermore, “relationships” were often valued at the end of life. Good relationships were considered promoting several themes in this map, which leads to protecting dignity and promoting personhood.
